# Supplementary material for: Hot spot prediction in protein-protein interactions by an ensemble system
Source: BMC Syst Biol. 2018 Dec 31;12(Suppl 9):132. doi: 10.1186/s12918-018-0665-8 (PMC6311905; doi:10.1186/s12918-018-0665-8)
Supplement: Supplementary file 2 — All relevant MATLAB code. A simple Matlab implement of our predictor. (ZIP 19,742 kb) [file 12918_2018_665_MOESM2_ESM.zip › Additional file 2/Read ASA/ASA website/ASEdb/1AHWC.htm]

 NetSurfP - Protein Surface Accessibility and Secondary Structure Predictions

  

|  |  |  |
| --- | --- | --- |
|  |  | NetSurfP - Protein Surface Accessibility and Secondary Structure Predictions  Technical University of Denmark |

  


---

```
# For publication of results, please cite:
# A generic method for assignment of reliability scores applied to solvent accessibility predictions.
# Bent Petersen, Thomas Nordahl Petersen, Pernille Andersen, Morten Nielsen and Claus Lundegaard
# BMC Structural Biology 2009, 9:51 doi:10.1186/1472-6807-9-51
#
# Column 1: Class assignment - B for buried or E for Exposed - Threshold: 25% exposure, but not based on RSA
# Column 2: Amino acid
# Column 3: Sequence name
# Column 4: Amino acid number
# Column 5: Relative Surface Accessibility - RSA
# Column 6: Absolute Surface Accessibility
# Column 7: Z-fit score for RSA prediction
# Column 8: Probability for Alpha-Helix
# Column 9: Probability for Beta-strand
# Column 10: Probability for Coil
E S  1AHWC                  1    0.843  98.788  -0.380   0.003   0.003   0.994
E G  1AHWC                  2    0.596  46.874  -2.102   0.018   0.047   0.935
E T  1AHWC                  3    0.537  74.510  -0.256   0.018   0.047   0.935
E T  1AHWC                  4    0.688  95.356  -0.551   0.018   0.088   0.893
E N  1AHWC                  5    0.668  97.766   0.112   0.018   0.047   0.935
B T  1AHWC                  6    0.130  17.976   0.440   0.018   0.047   0.935
E V  1AHWC                  7    0.349  53.657   0.205   0.018   0.019   0.964
E A  1AHWC                  8    0.458  50.505   0.863   0.018   0.019   0.964
B A  1AHWC                  9    0.162  17.830  -1.037   0.005   0.045   0.951
E Y  1AHWC                 10    0.442  94.498  -0.204   0.004   0.197   0.799
E N  1AHWC                 11    0.412  60.361  -0.180   0.004   0.420   0.576
B L  1AHWC                 12    0.046   8.478   0.634   0.003   0.718   0.279
E T  1AHWC                 13    0.273  37.921   1.412   0.001   0.900   0.099
B W  1AHWC                 14    0.036   8.658   0.449   0.001   0.959   0.040
E K  1AHWC                 15    0.248  51.055   1.459   0.001   0.959   0.040
B S  1AHWC                 16    0.031   3.680  -1.292   0.001   0.959   0.040
E T  1AHWC                 17    0.262  36.367   0.479   0.001   0.900   0.099
E N  1AHWC                 18    0.290  42.515  -1.103   0.004   0.197   0.799
B F  1AHWC                 19    0.155  31.048  -0.166   0.004   0.085   0.910
E K  1AHWC                 20    0.382  78.495   0.330   0.004   0.514   0.481
B T  1AHWC                 21    0.088  12.233   0.433   0.001   0.900   0.099
B I  1AHWC                 22    0.090  16.576   1.064   0.001   0.959   0.040
B L  1AHWC                 23    0.023   4.248   1.043   0.001   0.959   0.040
B E  1AHWC                 24    0.227  39.639   0.965   0.001   0.959   0.040
B W  1AHWC                 25    0.045  10.726  -0.537   0.002   0.816   0.182
E E  1AHWC                 26    0.428  74.754   0.975   0.004   0.420   0.576
E P  1AHWC                 27    0.302  42.854  -0.527   0.004   0.138   0.858
E K  1AHWC                 28    0.588 120.972  -0.256   0.005   0.045   0.951
E P  1AHWC                 29    0.519  73.632  -0.797   0.005   0.015   0.979
E V  1AHWC                 30    0.540  82.937  -1.504   0.005   0.015   0.979
E N  1AHWC                 31    0.550  80.447  -1.296   0.004   0.085   0.910
B Q  1AHWC                 32    0.168  30.005   0.282   0.004   0.616   0.381
B V  1AHWC                 33    0.231  35.535   0.747   0.001   0.900   0.099
B Y  1AHWC                 34    0.063  13.399   0.942   0.001   0.959   0.040
B T  1AHWC                 35    0.127  17.643   0.733   0.001   0.959   0.040
B V  1AHWC                 36    0.030   4.642   0.571   0.001   0.959   0.040
B Q  1AHWC                 37    0.127  22.700   0.335   0.001   0.959   0.040
B I  1AHWC                 38    0.101  18.685   0.348   0.001   0.959   0.040
B S  1AHWC                 39    0.215  25.163   0.686   0.001   0.959   0.040
E T  1AHWC                 40    0.395  54.814  -0.487   0.002   0.816   0.182
E K  1AHWC                 41    0.456  93.799  -0.741   0.021   0.279   0.699
E S  1AHWC                 42    0.594  69.593  -1.210   0.018   0.088   0.893
E G  1AHWC                 43    0.630  49.597   0.025   0.019   0.141   0.840
E D  1AHWC                 44    0.573  82.526   0.918   0.005   0.262   0.733
B W  1AHWC                 45    0.135  32.443   0.529   0.023   0.655   0.322
E K  1AHWC                 46    0.490 100.834   0.923   0.018   0.846   0.136
E S  1AHWC                 47    0.492  57.604   0.430   0.021   0.756   0.223
E K  1AHWC                 48    0.414  85.098   0.209   0.022   0.552   0.426
E C  1AHWC                 49    0.544  76.378  -1.636   0.021   0.451   0.528
E F  1AHWC                 50    0.407  81.785   0.408   0.022   0.359   0.619
E Y  1AHWC                 51    0.507 108.303  -0.443   0.020   0.205   0.775
B T  1AHWC                 52    0.196  27.157   0.226   0.019   0.141   0.840
E T  1AHWC                 53    0.517  71.639  -0.563   0.021   0.279   0.699
E D  1AHWC                 54    0.443  63.822   0.135   0.022   0.359   0.619
E T  1AHWC                 55    0.284  39.349   0.122   0.021   0.451   0.528
E E  1AHWC                 56    0.443  77.340  -0.240   0.023   0.655   0.322
B C  1AHWC                 57    0.054   7.554   0.492   0.022   0.552   0.426
E D  1AHWC                 58    0.309  44.512  -0.515   0.022   0.552   0.426
B L  1AHWC                 59    0.079  14.373  -0.085   0.022   0.359   0.619
B T  1AHWC                 60    0.116  16.034  -0.612   0.066   0.296   0.638
B D  1AHWC                 61    0.248  35.708  -1.155   0.216   0.235   0.548
E E  1AHWC                 62    0.409  71.417  -0.346   0.199   0.152   0.649
B I  1AHWC                 63    0.271  50.227  -0.720   0.199   0.152   0.649
E V  1AHWC                 64    0.318  48.877  -0.367   0.118   0.150   0.732
E K  1AHWC                 65    0.527 108.486  -0.630   0.052   0.084   0.864
E D  1AHWC                 66    0.427  61.588  -0.337   0.018   0.047   0.935
B V  1AHWC                 67    0.148  22.717   0.203   0.018   0.088   0.893
E K  1AHWC                 68    0.418  86.024  -0.656   0.005   0.262   0.733
B Q  1AHWC                 69    0.202  36.024  -0.683   0.004   0.616   0.381
B T  1AHWC                 70    0.210  29.155   0.951   0.001   0.900   0.099
B Y  1AHWC                 71    0.039   8.441   0.133   0.001   0.959   0.040
B L  1AHWC                 72    0.123  22.466   0.624   0.001   0.959   0.040
B A  1AHWC                 73    0.018   2.028   0.765   0.001   0.959   0.040
B R  1AHWC                 74    0.067  15.229   0.711   0.001   0.959   0.040
B V  1AHWC                 75    0.018   2.797   1.194   0.001   0.959   0.040
B F  1AHWC                 76    0.162  32.554   1.427   0.001   0.959   0.040
B S  1AHWC                 77    0.026   3.082  -0.393   0.001   0.959   0.040
B Y  1AHWC                 78    0.199  42.505   0.052   0.001   0.900   0.099
E P  1AHWC                 79    0.293  41.563  -0.145   0.004   0.514   0.481
E A  1AHWC                 80    0.416  45.821  -1.519   0.005   0.045   0.951
E G  1AHWC                 81    0.685  53.894  -2.577   0.018   0.019   0.964
E N  1AHWC                 82    0.478  69.994  -1.102   0.019   0.141   0.840
E V  1AHWC                 83    0.387  59.513  -1.039   0.022   0.359   0.619
E E  1AHWC                 84    0.302  52.724  -1.510   0.021   0.451   0.528
E S  1AHWC                 85    0.385  45.087  -1.363   0.022   0.359   0.619
E T  1AHWC                 86    0.442  61.250  -1.052   0.021   0.279   0.699
E G  1AHWC                 87    0.376  29.575  -1.550   0.021   0.279   0.699
E S  1AHWC                 88    0.476  55.740  -1.399   0.021   0.279   0.699
E A  1AHWC                 89    0.453  49.899  -1.409   0.021   0.279   0.699
E G  1AHWC                 90    0.505  39.775  -0.968   0.022   0.359   0.619
E E  1AHWC                 91    0.483  84.310  -0.997   0.021   0.279   0.699
B P  1AHWC                 92    0.292  41.463  -0.534   0.019   0.141   0.840
E L  1AHWC                 93    0.385  70.512  -0.515   0.019   0.141   0.840
B Y  1AHWC                 94    0.223  47.719  -0.453   0.021   0.279   0.699
E E  1AHWC                 95    0.318  55.624  -1.145   0.022   0.552   0.426
E N  1AHWC                 96    0.482  70.536  -0.938   0.021   0.451   0.528
B S  1AHWC                 97    0.164  19.174  -0.699   0.020   0.205   0.775
E P  1AHWC                 98    0.591  83.849  -0.934   0.019   0.141   0.840
E E  1AHWC                 99    0.435  75.995   0.293   0.020   0.205   0.775
B F  1AHWC                100    0.068  13.668  -1.026   0.022   0.359   0.619
E T  1AHWC                101    0.290  40.251   0.546   0.021   0.451   0.528
B P  1AHWC                102    0.178  25.301  -0.538   0.022   0.359   0.619
B Y  1AHWC                103    0.223  47.676   0.106   0.021   0.279   0.699
E L  1AHWC                104    0.468  85.654   0.132   0.021   0.279   0.699
E E  1AHWC                105    0.527  92.067  -0.722   0.021   0.279   0.699
B T  1AHWC                106    0.082  11.415  -0.169   0.021   0.451   0.528
E N  1AHWC                107    0.428  62.630   1.171   0.022   0.552   0.426
B L  1AHWC                108    0.059  10.876  -0.091   0.021   0.451   0.528
E G  1AHWC                109    0.309  24.295  -0.284   0.004   0.085   0.910
E Q  1AHWC                110    0.301  53.741  -0.030   0.005   0.015   0.979
B P  1AHWC                111    0.202  28.721  -1.001   0.005   0.045   0.951
E T  1AHWC                112    0.537  74.482   0.150   0.005   0.336   0.660
B I  1AHWC                113    0.155  28.675  -0.166   0.002   0.816   0.182
E Q  1AHWC                114    0.494  88.318   0.842   0.001   0.900   0.099
B S  1AHWC                115    0.254  29.769  -0.222   0.001   0.959   0.040
E F  1AHWC                116    0.325  65.167   0.692   0.001   0.959   0.040
E E  1AHWC                117    0.403  70.439   0.257   0.001   0.900   0.099
E Q  1AHWC                118    0.376  67.154  -0.094   0.003   0.718   0.279
E V  1AHWC                119    0.539  82.890  -0.712   0.005   0.336   0.660
E G  1AHWC                120    0.695  54.689  -1.270   0.005   0.015   0.979
E T  1AHWC                121    0.344  47.713  -0.080   0.004   0.138   0.858
B K  1AHWC                122    0.225  46.365   0.437   0.001   0.900   0.099
B V  1AHWC                123    0.025   3.873   0.564   0.001   0.959   0.040
B N  1AHWC                124    0.181  26.469   1.353   0.001   0.959   0.040
B V  1AHWC                125    0.022   3.412   0.867   0.001   0.959   0.040
B T  1AHWC                126    0.167  23.135   0.726   0.001   0.959   0.040
B V  1AHWC                127    0.028   4.365   0.506   0.001   0.900   0.099
E E  1AHWC                128    0.367  64.028   0.794   0.003   0.718   0.279
B D  1AHWC                129    0.170  24.468   0.181   0.004   0.197   0.799
B E  1AHWC                130    0.190  33.176  -0.862   0.018   0.047   0.935
E R  1AHWC                131    0.381  87.341  -0.578   0.018   0.088   0.893
E T  1AHWC                132    0.407  56.409  -0.632   0.052   0.084   0.864
B L  1AHWC                133    0.200  36.638  -1.170   0.064   0.216   0.721
B V  1AHWC                134    0.292  44.834  -1.047   0.064   0.216   0.721
E R  1AHWC                135    0.512 117.134  -0.533   0.056   0.142   0.802
E R  1AHWC                136    0.526 120.431  -1.488   0.052   0.084   0.864
E N  1AHWC                137    0.646  94.574  -1.344   0.053   0.043   0.903
E N  1AHWC                138    0.611  89.406  -1.899   0.053   0.043   0.903
E T  1AHWC                139    0.522  72.360  -0.606   0.064   0.216   0.721
E F  1AHWC                140    0.425  85.358  -0.412   0.021   0.451   0.528
B L  1AHWC                141    0.212  38.817   0.123   0.022   0.552   0.426
B S  1AHWC                142    0.249  29.183  -0.354   0.023   0.655   0.322
B L  1AHWC                143    0.097  17.761  -0.508   0.023   0.655   0.322
E R  1AHWC                144    0.334  76.372  -0.483   0.023   0.655   0.322
B D  1AHWC                145    0.241  34.786  -2.253   0.021   0.756   0.223
B V  1AHWC                146    0.177  27.190  -0.730   0.021   0.756   0.223
B F  1AHWC                147    0.213  42.849  -0.340   0.022   0.552   0.426
E G  1AHWC                148    0.411  32.314  -0.566   0.019   0.141   0.840
E K  1AHWC                149    0.515 105.853  -1.518   0.005   0.045   0.951
E D  1AHWC                150    0.580  83.650  -0.385   0.004   0.138   0.858
B L  1AHWC                151    0.115  21.020  -0.171   0.004   0.514   0.481
E I  1AHWC                152    0.304  56.185   1.043   0.001   0.900   0.099
B Y  1AHWC                153    0.038   8.121  -0.023   0.001   0.959   0.040
B T  1AHWC                154    0.175  24.314   1.047   0.001   0.959   0.040
B L  1AHWC                155    0.020   3.625   1.130   0.000   0.983   0.017
B Y  1AHWC                156    0.121  25.751   1.294   0.000   0.983   0.017
B Y  1AHWC                157    0.031   6.667   0.452   0.000   0.983   0.017
B W  1AHWC                158    0.196  47.018   0.529   0.000   0.983   0.017
B K  1AHWC                159    0.244  50.294   0.601   0.001   0.959   0.040
E S  1AHWC                160    0.520  60.956   0.157   0.005   0.336   0.660
E S  1AHWC                161    0.491  57.498  -0.608   0.005   0.045   0.951
E S  1AHWC                162    0.687  80.516  -0.143   0.004   0.085   0.910
E S  1AHWC                163    0.701  82.145   0.754   0.004   0.138   0.858
E G  1AHWC                164    0.457  35.997   0.290   0.004   0.514   0.481
E K  1AHWC                165    0.501 103.138   0.670   0.001   0.900   0.099
E K  1AHWC                166    0.424  87.114   0.571   0.001   0.959   0.040
E T  1AHWC                167    0.509  70.668   0.455   0.001   0.959   0.040
E A  1AHWC                168    0.373  41.138  -0.281   0.001   0.900   0.099
E K  1AHWC                169    0.405  83.267  -0.475   0.004   0.616   0.381
E T  1AHWC                170    0.481  66.784  -0.882   0.004   0.420   0.576
E N  1AHWC                171    0.606  88.704  -0.422   0.005   0.262   0.733
E T  1AHWC                172    0.286  39.654  -0.409   0.004   0.420   0.576
E N  1AHWC                173    0.488  71.458   0.011   0.003   0.718   0.279
E E  1AHWC                174    0.322  56.306   0.484   0.001   0.900   0.099
B F  1AHWC                175    0.221  44.435   0.524   0.001   0.900   0.099
B L  1AHWC                176    0.123  22.466   0.173   0.018   0.846   0.136
E I  1AHWC                177    0.409  75.647  -0.074   0.004   0.616   0.381
E D  1AHWC                178    0.524  75.523  -0.112   0.004   0.420   0.576
B V  1AHWC                179    0.060   9.283   0.000   0.004   0.138   0.858
E D  1AHWC                180    0.509  73.304   0.894   0.005   0.015   0.979
E K  1AHWC                181    0.542 111.572  -0.081   0.005   0.015   0.979
E G  1AHWC                182    0.572  45.016  -0.868   0.003   0.003   0.994
B E  1AHWC                183    0.177  30.869  -0.020   0.004   0.197   0.799
E N  1AHWC                184    0.304  44.579   0.044   0.003   0.718   0.279
B Y  1AHWC                185    0.073  15.536   0.973   0.001   0.900   0.099
B C  1AHWC                186    0.029   4.015   0.416   0.001   0.959   0.040
B F  1AHWC                187    0.031   6.322   0.446   0.001   0.959   0.040
B S  1AHWC                188    0.151  17.756   0.997   0.001   0.959   0.040
B V  1AHWC                189    0.030   4.550   0.474   0.001   0.959   0.040
B Q  1AHWC                190    0.189  33.720   1.004   0.001   0.959   0.040
B A  1AHWC                191    0.047   5.212  -0.783   0.001   0.900   0.099
B V  1AHWC                192    0.149  22.932   0.107   0.001   0.959   0.040
B I  1AHWC                193    0.077  14.264   0.384   0.002   0.816   0.182
B P  1AHWC                194    0.203  28.820  -0.286   0.021   0.279   0.699
B S  1AHWC                195    0.230  26.991  -1.306   0.019   0.141   0.840
E R  1AHWC                196    0.443 101.539  -0.798   0.052   0.084   0.864
E T  1AHWC                197    0.452  62.734  -1.461   0.052   0.084   0.864
E V  1AHWC                198    0.409  62.817  -0.988   0.053   0.043   0.903
E N  1AHWC                199    0.603  88.235  -2.324   0.053   0.043   0.903
E R  1AHWC                200    0.459 105.180  -0.337   0.018   0.047   0.935
E K  1AHWC                201    0.501 102.973  -0.373   0.018   0.088   0.893
B S  1AHWC                202    0.188  21.987  -1.324   0.018   0.047   0.935
E T  1AHWC                203    0.638  88.477  -0.436   0.018   0.047   0.935
E D  1AHWC                204    0.414  59.600  -0.703   0.018   0.019   0.964
B S  1AHWC                205    0.131  15.412  -1.199   0.018   0.019   0.964
E P  1AHWC                206    0.517  73.348   0.694   0.053   0.043   0.903
E V  1AHWC                207    0.470  72.178   0.053   0.021   0.279   0.699
E E  1AHWC                208    0.419  73.164   1.833   0.021   0.756   0.223
B C  1AHWC                209    0.052   7.245  -0.080   0.011   0.918   0.071
E M  1AHWC                210    0.282  56.488   0.761   0.011   0.918   0.071
E G  1AHWC                211    0.546  43.002   0.174   0.002   0.816   0.182
B Q  1AHWC                212    0.224  40.060   1.212   0.004   0.420   0.576
E E  1AHWC                213    0.535  93.395  -0.223   0.004   0.085   0.910
E K  1AHWC                214    0.750 154.337   0.332   0.005   0.015   0.979
E G  1AHWC                215    0.451  35.478  -1.877   0.018   0.047   0.935
E E  1AHWC                216    0.585 102.112  -0.953   0.018   0.088   0.893
E F  1AHWC                217    0.325  65.147  -1.803   0.018   0.047   0.935
E R  1AHWC                218    0.640 146.583  -0.236   0.018   0.019   0.964
E E  1AHWC                219    0.895 156.426  -0.892   0.003   0.003   0.994

Explain the output.  Go back.
```

---
